# ﻿Two new species and new records of the genus *Procirrus* Latreille from China (Coleoptera, Staphylinidae, Paederinae)

**DOI:** 10.3897/zookeys.1250.158452

**Published:** 2025-08-27

**Authors:** Lei Huang, Guang-Hong Cao, Zhong Peng

**Affiliations:** 1 Department of Biology, Shanghai Normal University, Shanghai, 200234, China Shanghai Normal University Shanghai China; 2 Administration of Nabanhe River Watershed National Nature Reserve, Jinghong, 666100, China Administration of Nabanhe River Watershed National Nature Reserve Jinghong China

**Keywords:** China, morphology, new records, new species, rove beetles, taxonomic key

## Abstract

New taxonomic and faunistic data for three species of the genus *Procirrus* Latreille, 1829 from China are provided. Two new species from Hainan and Yunnan provinces are described and illustrated: *P. hei* Huang & Peng, **sp. nov.** and *P. yunnanensis* Huang & Peng, **sp. nov.** Additional data (including photographs of the habitus and the type labels) on the type specimen of *P. fusculus* Sharp, 1889 are given. *Procirrus lewisii* Sharp, 1889 is recorded from Shandong, Jiangsu, Shanghai, Zhejiang, Fujian, Hubei, Guangxi, Guizhou, Chongqing, Sichuan and Xizang for the first time. An identification key to Asian species of *Procirrus* is given.

## ﻿Introduction

To date, 30 species of the genus *Procirrus* Latreille have been reported, of which there are six species from the Palearctic and Oriental regions: *P. feae* Fauvel, 1895, *P. fusculus* Sharp, 1889, *P. hermani* Drugmand, 1989, *P. lefebvrei* Latreille, 1829, *P. lewisii* Sharp, 1889, and *P. saulcyi* Fauvel, 1873 (Newton 2025). The Ethiopian region is known for having the greatest diversity of *Procirrus* species. Within Chinese territories, only *Procirrus lewisii* has been documented as the sole representative species of this genus (Xu et al. 2020).

A study of *Procirrus* material from China yielded two species new to science and additional records of *P. lewisii* Sharp, 1889. Additional data on the type of *P. fusculus* Sharp, 1889 and a key to Asian species of *Procirrus* are given.

## ﻿Material and methods

The material treated in this study is deposited in the following collections:

**BMNH**Natural History Museum, London (M.V.L. Barclay, M. Geiser & K. Matsumoto);

**SNUC** Insect Collection of Shanghai Normal University, Shanghai;

**cFen** private collection Yu-Cheng Feng, Nanjing;

**cGao** private collection Ming-Yang Gao, Jinan;

**cRua** private collection Yu-Feng Ruan, Liuzhou.

Photographs were taken with a Canon EOS 7D camera with a Canon MP-E 65 mm macro lens or with a Canon G12 camera mounted on an Olympus CX31 microscope.

The following abbreviations are used in the text, with all measurements in millimeters:

**Total length (TL)** length of body from anterior margin of head to abdominal apex.

**Length of forebody (FL)** length of forebody from anterior margin of head to posterior margin of elytra.

**Head length (HL)** length of head from anterior margin of frons to posterior constriction of head.

**Head width (HW)** maximum width of head.

**Antenna length (AnL)** length of antenna from the base to the apex.

**Pronotum length (PL)** length of pronotum along midline.

**Pronotum width (PW)** maximum width of pronotum.

**Elytral length (EL)** length at suture from apex of scutellum to elytral hind margin.

**Elytral width (EW)** combined width of elytra.

**Length of aedeagus (AL)** length of aedeagus from apex of dorsal plate to base of aedeagal capsule.

The type labels are cited in the original spelling.

## ﻿Results

### ﻿ Procirrus fusculus

Taxon classificationAnimaliaColeopteraStaphylinidae

Sharp, 1889

5D108EDD-06EE-5B2E-A33B-5082169B2797

Fig. 1

#### Type material.

***Lectotype*.** • 1 ex.; glued on card, a specimen with five labels as follows: “Type” “Ind. or. Dacca” “*Procirrus fusculus* Type D. S. Dacca. Ind. or.” “Sharp Coll. 1905-313.” “NHMUK014383008” (BMNH).

#### Comments.

**Figure 1.**
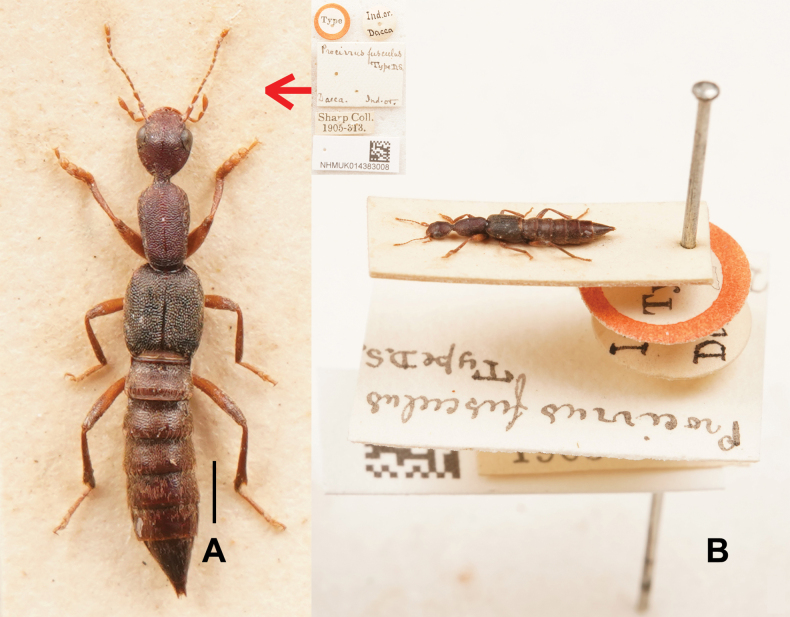
*Procirrus fusculus*. A. Habitus (Lectotype); B. Habitus and labels in lateral view (lectotype). Scale bars: 1.0 mm.

Habitus as in Fig. 1A, B. The original description is based on an unspecified number of syntypes, among them at least one specimen, from “Ind. or. Dacca” (Sharp 1889). *Procirrus fusculus* was redescribed in the Japanese literature by Tsunamitsu Adachi (1955) with concise descriptions, documenting its distribution in Japan and India (Japan: curated by Sharp; India: label transcription indicates “Ind. or. Dacca”). However, according to the type label, its distribution was exclusively reported from Bangladesh (Herman 2010). Through examination of the type specimen of *P. fusculus* and extensive comparative specimens of *P. lewisii* Sharp, 1889, the two species were found to share a few diagnostic characters in external morphology. However, due to the absence of a genitalic dissection and study of the type specimen of *P. fusculus*, conclusive determination of their conspecificity remains elusive.

### ﻿ Procirrus lewisii

Taxon classificationAnimaliaColeopteraStaphylinidae

Sharp, 1889

E2F72D11-B5CA-5F7C-B1DA-A4E5DDF3C735

Figs 2 , 3A , 4

#### Material examined.

China – **Shandong** • 1 ♂; Jinan, 03.VII.2023, Ming-Yang Gao leg. (cGao); China – **Jiangsu** • 1 ♂; Nanjing, Nanjing Agricultural University (Binjiang Campus), 27.II.2025, Yu-Cheng Feng leg. (cFen); China – **Shanghai** • 1 ♂; Chomgming, 21.IX.2018, Xiao-Bin Song leg. (SNUC); China – **Shanghai** • 1 ♀; Pudong New Area, Zhangjiang, 25.IV.2025, Liang Tang leg. (SNUC); China – **Zhengjiang** • 2 ♂♂; Jiande City, near Yuquan Road, alt. 50 m, 15.VII.2016, Yi-Yang Xu leg. (SNUC); China – **Zhengjiang** • 2 ♂♂, 1 ♀; Ningbo City, Mt. Tiantong, 26.IV.2009, Feng & Sheng leg. (SNUC); China – **Zhengjiang** • 1 ♂; Zhuji City, Dashanxia, alt. 100 m, 09.IX.2013, Tie-Xiong Zhao leg. (SNUC); China – **Zhengjiang** • 1 ♂; Jiangshan Co., Laofoyan Town, 27°55'03"N, 119°11'34"E, alt. 496 m, 11.VIII.2018, Cheng & Miao leg. (SNUC); China – **Fujian** • 1 ♀; Shaowu City, near Wanfu Temple, 27.35°N, 117.51°E, alt. 350 m, 06.VIII.2024, Jing-Han Chen leg. (SNUC); China – **Hubei** • 2 ♀♀; Wuhan City, Jinyinhu Park, 30°38'46"N, 114°10'52"E, alt. 5 m, 09.VII.2020, Zi-Hao Shen leg. (SNUC); China – **Guangdong** • 1 ♂; Shenzhen City, Mt. Qiniang, 22°32'31"N, 114°35'15"E, 0–91 m, 24.V.2023, Cai, Yan, Yin & Zhang leg. (SNUC); China – **Guangdong** • 1 ♂; Shenzhen City, Mt. Qiniang, 22°32'24"N, 114°35'24"E, 0–20 m, 27.V.2021, Cai & Zhou leg. (SNUC); China – **Guangdong** • 2 ♂♂; Shenzhen City, Mt. Qiniang, 22°32'29"N, 114°35'08"E, alt. 45 m, 24.III.2019, Tang, Shuai, Xia, Zhao & Zhou leg. (SNUC); China – **Guangdong** • 1 ♂; Shenzhen City, Mt. Qiniang, 22°32'29"N, 114°35'08"E, alt. 65 m, 08.VII.2019, Chang, Xia, Zhang, Zhao & Zhou leg. (SNUC); China – **Guangdong** • 1 ♂; Shenzhen City, Mt. Wutong, 22°35'43"N, 114°11'55"E, 0–600 m, 22.VII.2021, Pan & Zhou leg. (SNUC); China – **Guangdong** • 1 ♂; Shenzhen City, Mt. Wutong, 22°35'43"N, 114°11'55"E, alt. 100 m, 17.III.2021, Pan & Zhou leg. (SNUC); China – **Guangdong** • 1 ♂; Shenzhen City, Mt. Meilin, 22°32'31"N, 114°35'13"E, alt. 280 m, 09.VI.2019, Cai, Tang, Huang, Shuai & Zhao leg. (SNUC); China – **Guangdong** • 1 ♀; Shenzhen City, Mt. Meilin, 22°34'53"N, 114°02'58"E, alt. 20 m, 22.IX.2021, Cai & Zhou leg. (SNUC); China – **Guangxi** • 1 ♀; Jinxiu Co., 16 km, 850–950 m, 24.VII.2011, Zhong Peng leg. (SNUC); China – **Guangxi** • 1 ♂, 1 ♀; Longzhou Co., Nonggang N. R., alt. 200 m, 29.VI.2015, Lu Qiu leg. (SNUC); China – **Guangxi** • 1 ♀; Guilin City, Huaping N. R., 25°37'39"N, 109°54'20"E, alt. 520 m, 18.VIII.2020, Lu Qiu leg. (SNUC); China – **Guangxi** • 2 ♀♀; Longjiang Co., Daren Shan, 700–800 m, 16.I.2025, Yu-Feng Ruan leg. (cRua); China – **Guizhou** • 1 ♂; Rongjiang Co., Xiaodanjiang, 26°20'16"N, 108°20'23"E, alt. 700 m, 05.V.2021, Cai, Peng, Song & Tang leg. (SNUC); China – **Guizhou** • 1 ♀; Guiyang City, Huaxi, Shi-Li-He-Tang, 26°27'48"N, 106°40'36"E, alt. 1157 m, 25.IX.2021, Ri-Xin Jiang leg. (SNUC); China – **Chongqing** • 1 ♂; Aoki, 29.71°N, 106.29°E, alt. 485 m, 28.X.2017, Zhi-Zhong Gao leg. (SNUC); China – **Chongqing** • 1 ♂; Nanshan Botanical Garden, 29.55°N, 106.60°E, alt. 428 m, 22.X.2017, Zhi-Zhong Gao leg. (SNUC); China – **Chongqing** • 1 ♀; Beipei, Renhe Town, 29.78°N, 106.36°E, alt. 352 m, 19.X.2017, Zhi-Zhong Gao leg. (SNUC); China – **Sichuan** • 1 ♂; Gulin Co., Longping Town, 28°01'04"N, 105°48'11"E, alt. 950 m, 17.III.2020, Lu Qiu leg. (SNUC); China – **Xizang** • 1 ♂; Chayu Co., near Xiachayu Twon, 28°32'00"N, 96°58'41"E, alt. 1570 m, 30.VII.2019, Zi-Wei Yin leg; (SNUC); China – **Hong Kong** • 1 ♂; Mt. Taimo, c. 380 m, 05.IV.2013, Song & Yin leg; (SNUC).

**Figure 2.**
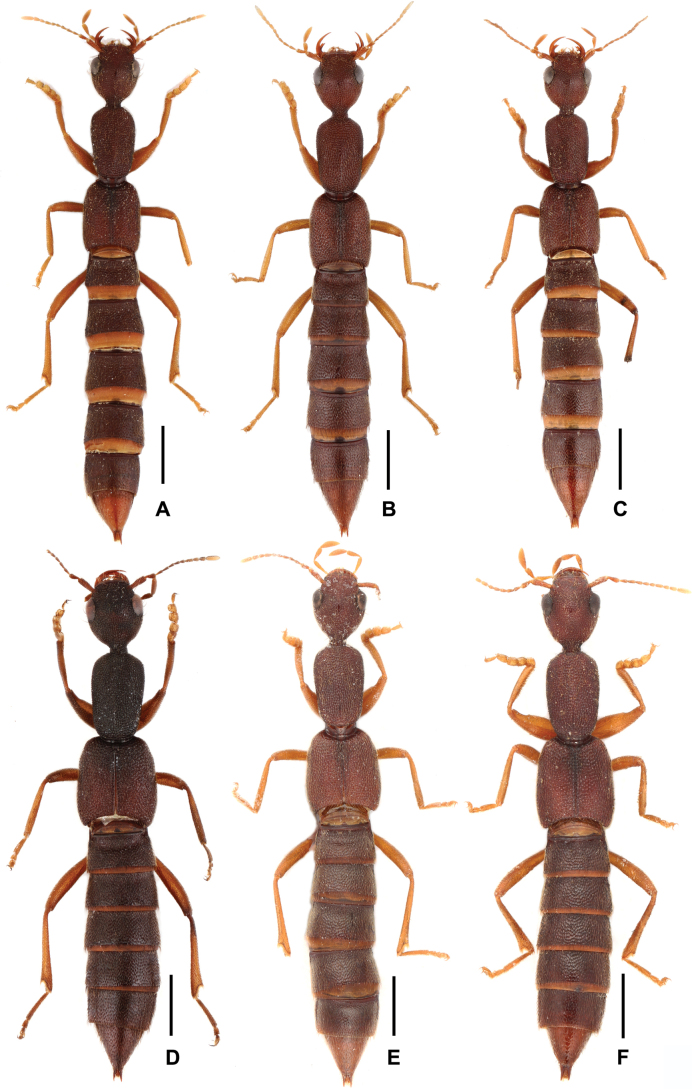
Habitus of *Procirrus lewisii* from different provinces. A. Zhejiang; B. Guangdong; C, D. Guangxi; E. Guizhou; F. Sichuan. Scale bars: 1.0 mm.

#### Comments.

Previously, this species was known from Japan, South Korea, Vietnam, Thailand and the Chinese provinces Guangdong, Hong Kong and from Taiwan (Xu et al. 2020; Senda et al. 2023). The aforementioned material indicates that this species is common and widespread throughout East Asia and the Oriental Realm, exhibiting considerable intraspecific variation in morphological traits. These include body size, coloration, punctation, and the shape of the head, pronotum and elytra, as well as internal structures of the aedeagus. However, minimal variation occurs in the aedeagus framework itself, and the shape of male sternite VIII remains constant. The above specimens from Shandong, Jiangsu, Shanghai, Zhejiang, Fujian, Hubei, Guangxi, Guizhou, Chongqing, Sichuan and Xizang represent new provincial records.

**Figure 3.**
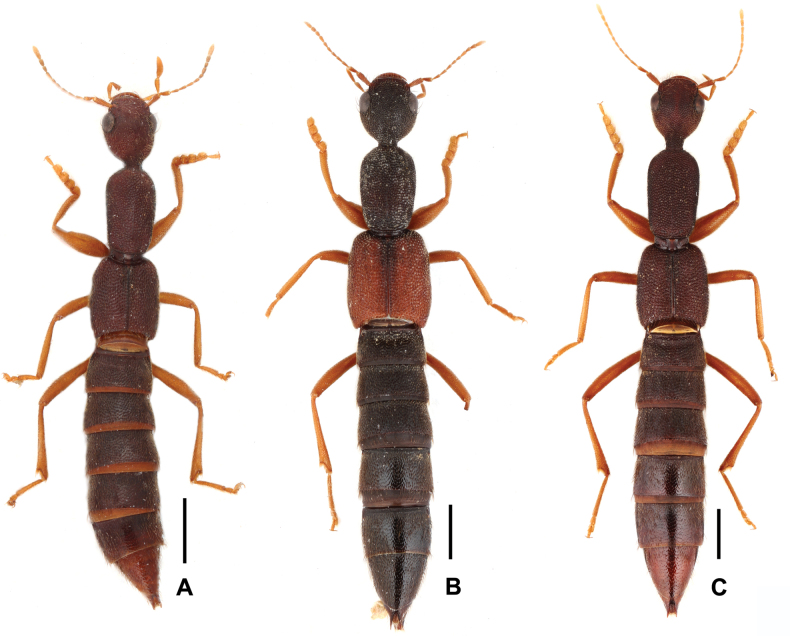
Habitus. A. *Procirrus lewisii* (from Xizang); B. *Procirrus hei*; C. *Procirrus yunnanensis*. Scale bars: 1.0 mm.

**Figure 4.**
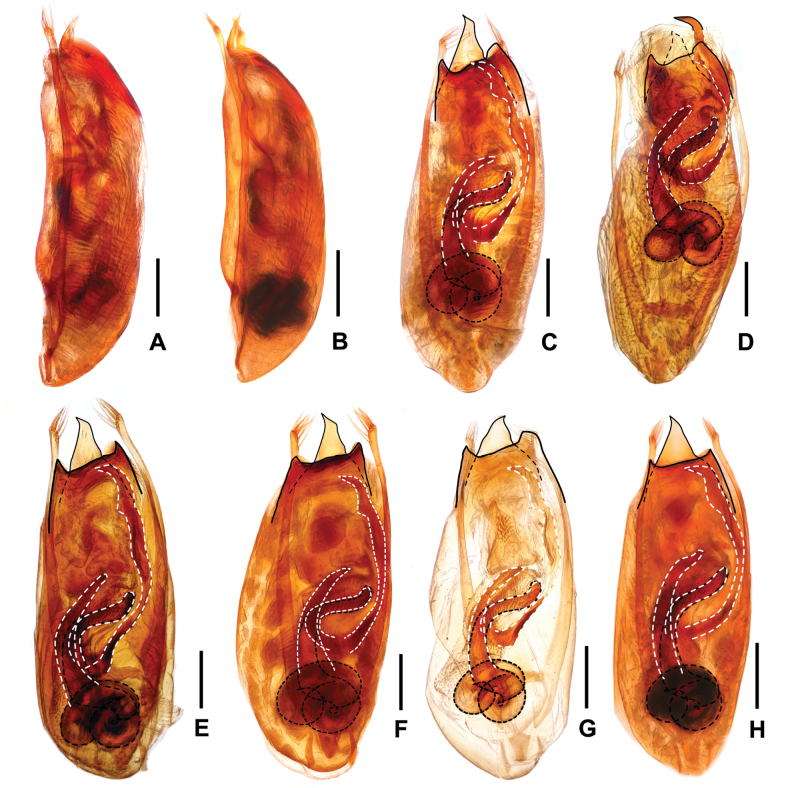
Aedeagus of *Procirrus lewisii* from different provinces. A, B. Lateral view: A. Zhejiang; B. Xizang; C–H. Ventral view: C. Zhejiang; D. Guangdong; E. Guangxi; F. Guizhou; G. Chongqing; H. Xizang. Scale bars: 0.2 mm.

### ﻿ Procirrus hei

Taxon classificationAnimaliaColeopteraStaphylinidae

L. Huang & Z. Peng sp. nov.

1D8A3C01-DE90-5ED9-80D3-14E78EDE9F60

https://zoobank.org/0EFFCB31-0A77-4CEF-843B-124C178B178C

Figs 3B , 5

#### Type material.

***Holotype*.** China – **Hainan Prov.** • ♂; glued on a card with two labels as follows: “China: Hainan Prov., Baisha Co., Yinggeling, alt. 200 m, 10.VIII.2009, Zhu-Qing He leg.” “HOLOTYPE: *Procirrus hei* sp. nov., Huang & Peng des. 2025” [red handwritten label]; (SNUC). ***Paratype*.** China – **Hainan Prov.**• 1 ♀; glued on a card with two labels as follows: “China: Hainan Prov., Baisha Co., Yinggeling, alt. 200 m, 10.VIII.2009, Zhu-Qing He leg.” “PARATYPE: *Procirrus hei* sp. nov., Huang & Peng des. 2025” [yellow handwritten label]; (SNUC).

#### Description.

Measurements (in mm) and ratios: TL: 9.20–10.10; FL: 4.39–4.61; HL: 1.08–1.11; HW: 1.08; AnL: 1.79–1.80; PL: 1.56–1.57; PW: 1.00–1.03; EL: 1.28–1.39; EW: 1.53–1.56; AL: 1.95; HW/HL: 0.97–1.00; HW/PW: 1.05–1.08; HL/PL: 0.69–0.71; PW/PL: 0.64–0.66; EL/PL: 0.82–0.89.

Habitus as in Fig. 3B. Coloration: head, pronotum and abdomen blackish-brown; elytra reddish; antennae and legs brown to light brown.

Head nearly orbicular, widest across eyes; punctation rather fine, weakly umbilicate and very dense, surface matt. Eyes large, 0.73 times as long as postocular region in dorsal view. Antennae slender, antennomere 1 2.17–3.08 times, 2 1.50–2.00 times, 3 1.17–1.62 times, 11 1.28–1.85 times as long as 4. Neck across nuchal constriction one-fourth to one-third as wide as greatest postocular width of head.

Pronotum distinctly oblong; lateral margins weakly convex in dorsal view; punctation similar to that of head, but coarser; midline with short and very narrow rudiment of glossy line posteriorly.

Elytra shorter than pronotum; humeral angles well developed; punctation weakly coarser and sparser than that of pronotum; pubescence denser than that of pronotum. Hind wings well developed. Protarsomeres 1–4 strongly dilated.

Abdomen parallel, widest at segment VI, evenly narrowing posteriorly. Abdominal tergites with coarse and dense punctation, and long decumbent pubescence, weakly denser on apical tergites; interstices without microsculpture.

**Figure 5.**
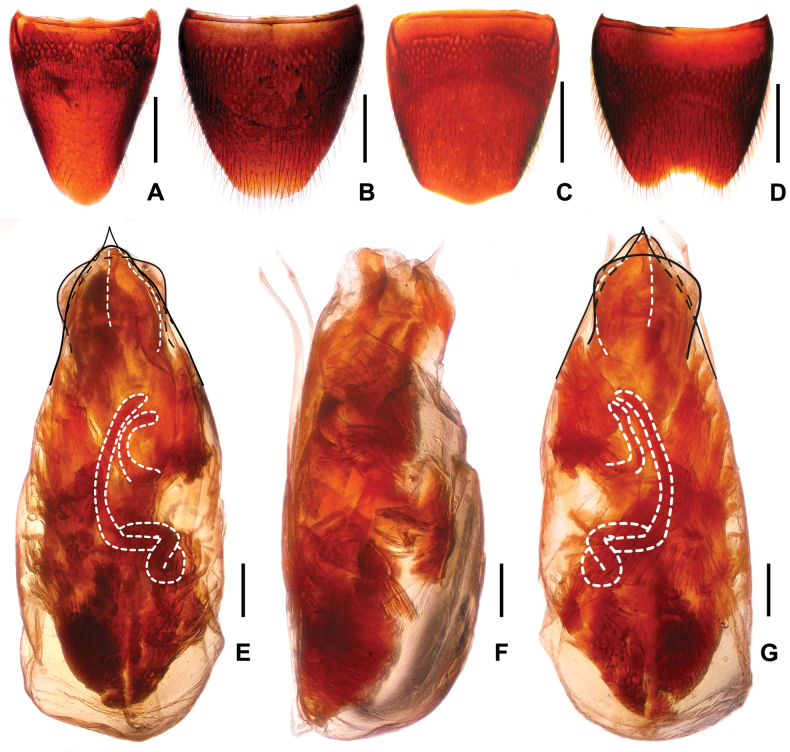
*Procirrus hei*. A. Female tergite VIII; B. Female sternite VIII; C. Male tergite VIII; D. Male sternite VIII; E. Aedeagus in ventral view; F. Aedeagus in lateral view; G. Aedeagus in dorsal view. Scale bars: 0.5 mm (A–D); 0.2 mm (E–G).

**Male.** Posterior margin of abdominal tergite VIII (Fig. 5C) convex. Sternite VIII (Fig. 5D) weakly transverse, with deep and weakly asymmetric posterior excision, pubescence not distinctly modified. Aedeagus as in Fig. 5E–G; median lobe blade-shaped in ventral view and somewhat curved in lateral view; parameres slender starting from base, with about 5–6 apical setae; internal sac with somewhat hook-shaped sclerotized spine basally and large sclerotized spine apically, and with long flagellum coiled in basal part of aedeagus.

**Female.** Abdominal tergite VIII (Fig. 5A) oblong, with strongly convex posterior margin. Posterior margin of abdominal sternite VIII (Fig. 5B) broadly convex.

#### Distribution and natural history.

The type locality is situated in Yinggeling to the east of Baisha, central Hainan. Two specimens were sifted from grass roots and humus in an evergreen broad-leaved forest at an altitude of 200 m.

#### Etymology.

This species is dedicated to Zhu-Qing He, who is a renowned specialist on mainly Palaearctic Orthoptera and the collector of the type specimens.

#### Comparative notes.

Regarding the general shape of the body, aedeagus, and male sternite VIII, *P. hei* is similar to dark-coloured specimens of morphologically variable *P. lewisii*, a widespread species in East Asia. It can be distinguished from it by the larger body size, the reddish elytra, the blade-shaped median lobe of aedeagus in ventral view and two sclerotized spines of different shapes in the internal sac (*P. lewisii*: median lobe asymmetric and moderately concave apically; internal sac with three sclerotized spines).

### ﻿ Procirrus yunnanensis

Taxon classificationAnimaliaColeopteraStaphylinidae

L. Huang & Z. Peng sp. nov.

7965F19E-8A6B-5ACA-8A20-E1A6F1006CD6

https://zoobank.org/E70A74AA-6A9E-41D6-9C9A-B0F291C38306

Figs 3C , 6

#### Type material.

***Holotype*.** China – **Yunnan Prov.** • ♂; glued on a card with two labels as follows: “China: Yunnan Prov., Lingcang City, Yun Co., Manwan Town, Waziba Village, 24°43'48"N, 100°20'01"E, alt. 1243 m, 16.IV.2016, Zi-Chun Xiong leg.” “HOLOTYPE: *Procirrus yunnanensis* sp. nov., Huang & Peng des. 2025” [red handwritten label]; (SNUC). ***Paratypes*.** China – **Yunnan Prov.** • 1 ♂; glued on a card with two labels as follows: “China: Yunnan Prov., Xishuangbanna, Botanical Garden of the Chinese Academy of Sciences, 5.VII.2003, Hu & Tang leg.” “PARATYPE: *Procirrus yunnanensis* sp. nov., Huang & Peng des. 2025” [yellow handwritten label]; (SNUC); China – **Yunnan Prov.** • 1 ♀; glued on a card with two labels as follows: “China: Yunnan Prov., Xishuangbanna, Nabanhe, alt. 700 m, 5.V.2009, Hu & Yin leg.” “PARATYPE: *Procirrus yunnanensis* sp. nov., Huang & Peng des. 2025” [yellow handwritten label]; (SNUC).

#### Description.

Measurements (in mm) and ratios: TL: 9.26–10.72; FL: 4.84–4.89; HL: 1.20–1.22; HW: 1.03–1.06; AnL: 2.14–2.17; PL: 1.67–1.75; PW: 0.97–1.00; EL: 1.11; EW: 1.28–1.36; AL: 2.00–2.67; HW/HL: 0.86–0.88; HW/PW: 1.06–1.09; HL/PL: 0.69–0.72; PW/PL: 0.55–0.58; EL/PL: 0.63–0.66.

Habitus as in Fig. 3C. Coloration: body brown; antennae brown to yellowish brown; legs brown to light brown.

Head nearly orbicular, widest behind eyes; punctation fine and very dense, surface matt. Eyes large, 0.81 times as long as postocular region in dorsal view. Antennae slender, antennomere 1 2.14–2.25 times, 2 1.36–1.45 times, 3 0.95–1.09 times, 11 1.23–1.50 times as long as 4. Neck across nuchal constriction one-third as wide as greatest postocular width of head.

Pronotum distinctly oblong; lateral margins weakly convex in dorsal view; coarser and denser than that of head; midline with short and very narrow rudiment of glossy line posteriorly.

Elytra shorter than pronotum; humeral angles well developed; punctation coarser and sparser than that of pronotum; pubescence denser than that of pronotum. Hind wings well developed. Protarsomeres I–IV strongly dilated.

Abdomen nearly parallel, widest at segment VI, evenly narrowing posteriorly. Abdominal tergites with coarse and dense punctation, and long decumbent pubescence, distinctly denser on apical tergites; interstices without microsculpture.

**Figure 6.**
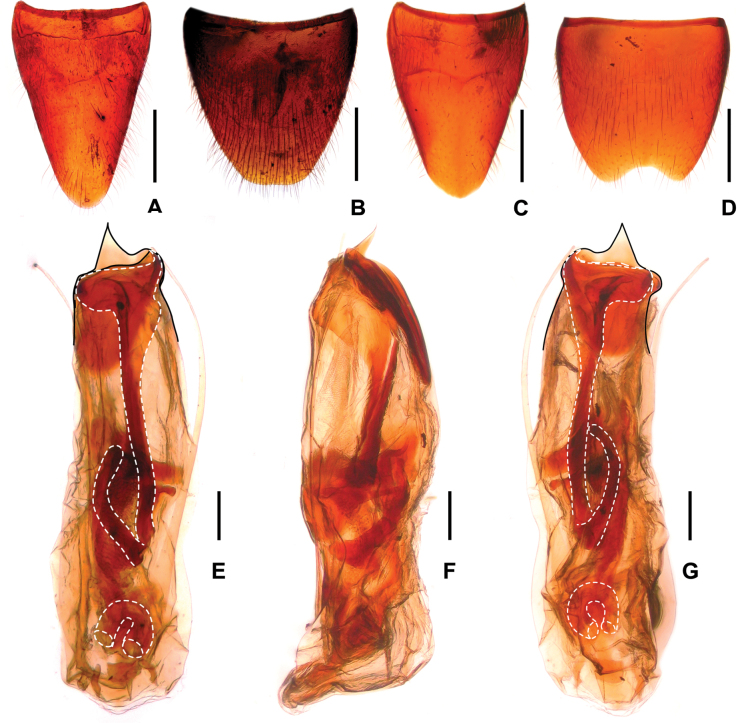
*Procirrus yunnanensis*. A. Female tergite VIII; B. Female sternite VIII; C. Male tergite VIII; D. Male sternite VIII; E. Aedeagus in ventral view; F. Aedeagus in lateral view; G. Aedeagus in dorsal view. Scale bars: 0.5 mm (A–D); 0.2 mm (E–G).

**Male.** Abdominal tergite VIII (Fig. 6C) oblong, with strongly convex posterior margin. Sternite VIII (Fig. 6D) weakly transverse, with moderately deep and weakly asymmetric posterior excision, pubescence not distinctly modified. Aedeagus slender and as in Fig. 6E–G; median lobe asymmetric in ventral view; parameres slender starting from base, with about 1–2 apical setae; internal sac with somewhat hook-shaped sclerotized spine basally and large sclerotized spine apically, and with short flagellum curled in basal part of aedeagus.

**Female.** Abdominal tergite VIII (Fig. 6A) distinctly oblong, with strongly convex posterior margin. Posterior margin of abdominal sternite VIII (Fig. 6B) broadly convex.

#### Distribution and natural history.

The specimens were collected in three localities in southwest Yunnan, China. They were sifted from the leaf litter of broad-leaved forests.

#### Etymology.

The specific epithet is derived from Yunnan Province, where the type locality is situated.

#### Comparative notes.

Regarding the coloration of the body, the shape of the pronotum, the elytra and the male sternite VIII, as well as features of the punctation and pubescence, *P. yunnanensis* is similar to light-coloured specimens of morphologically variable *P. lewisii*. It can be distinguished from it by the larger body size, the slightly longer neck, the slender aedeagus with two sclerotized spines and a short flagellum curled in the basal part of the internal sac (*P. lewisii*: internal sac with three sclerotized spines and a long flagellum).

### ﻿Key to the *Procirrus* species of Asia

**Table:** 

1	Micropterous species, elytra nearly trapezoidal. Israel, Lebanon, Turkey; Iran?	***P. saulcyi* Fauvel, 1873**
–	Macropterous species, elytra nearly parallel	**2**
2	Pronotum widest near anterior fourth, lateral margins converging posteriorly in dorsal view	**3**
–	Pronotum widest near anterior third to half, lateral margins weakly convex in dorsal view	**4**
3	Elytra slightly wider than the pronotum, punctuation slightly finer than that of the pronotum. Israel	***P. hermani* Drugmand, 1989**
–	Elytra obviously wider than the pronotum, punctuation obviously coarser than that of the pronotum. India, Myanmar	***P. feae* Fauvel, 1895**
4	Larger species, length of body at least 9.3 mm	**5**
–	Smaller species, length of body at most 8.7 mm	**6**
5	Head, pronotum and abdomen blackish-brown. Aedeagus stout; parameres with 5–6 apical setae. China	***P. hei* L. Huang & Z. Peng, sp. nov.**
–	Coloration of body brown. Aedeagus slender; parameres with 1–2 apical setae. China	***P. yunnanensis* L. Huang & Z. Peng, sp. nov.**
6	Elytra blackish-brown. Femora dark-brown. Bangladesh	***P. fusculus* Sharp, 1889**
–	Elytra reddish-brown to brown. Femora reddish-brown to brown. Japan, South Korea, Vietnam, Thailand, China	***P. lewisii* Sharp, 1889**

## Supplementary Material

XML Treatment for Procirrus fusculus

XML Treatment for Procirrus lewisii

XML Treatment for Procirrus hei

XML Treatment for Procirrus yunnanensis

## ﻿Additional information

### Use of AI

No use of AI was reported.

### Author ORCIDs

Zhong Peng https://orcid.org/0000-0001-5959-1536

### Data availability

All of the data that support the findings of this study are available in the main text.
